# Space for STEAM: New Creativity Challenge in Education

**DOI:** 10.3389/fpsyg.2021.586318

**Published:** 2021-03-23

**Authors:** Henderika de Vries

**Affiliations:** Yale Center for Emotional Intelligence, Yale Child Study Center, Yale University, New Haven, CT, United States

**Keywords:** space, STEAM, scientific creativity, culture, teaching practices, creative cognition

## Introduction

Governments recognized that a sustainable future requires solving new problems of a rapidly changing world in an innovative and interdisciplinary way. The importance to prepare learners for innovative thinking is for example expressed in educational goals (OECD Education 2030; UN 2030 Global Goals for Sustainable Development (SDGs), 2018).

STEM (Science, Technology, Engineering, and Mathematics) education first came about through development in the field of education, which realized that not only content, but higher order thinking is needed (De Boer, 1991; Sanders et al., 2011). Further, global evolution in education took place, and pedagogies emerged to engage all students in STEM fields. Art was added and thought to engage students, foster inclusive and gender equal classrooms, and therefore helping to achieve success and promote critical and creative thinking of all students (Bae et al., 2014; Harris and de Bruin, 2017). This resulted in an integration of the creative arts within the scientific and technical disciplines, STEAM (Science, Technology, Engineering, Arts, Mathematics).

However, questions can be raised how and if educational goals are achieved. For example, today's classrooms are increasingly multicultural, requiring an understanding of cultural differences in teaching practices as part of intercultural competence of teachers (e.g., Wursten and Jacobs, 2013; Thapa, 2020). Moreover, although there is an increase in empirical studies (for an overview, see Saptono and Hidayah, 2020), many reasoning processes of STEM education, particularly those pertaining to scientific creative reasoning, are still not well understood (Sternberg et al., 2020). Some studies find social (de Vries and Lubart, 2017) or cross-cultural aspects related to scientific creative cognition (De Vries, 2018). These results indicate that there might be cultural factors related to STEAM teaching as well, which are unknown today. Research studies on STEAM education are largely qualitative (e.g., Barlex and Pitt, 2000; Keys and Bryan, 2001), and integration of findings from empirical research with qualitative research on teaching practices is rare.

Overall, there is a gap within the STEAM framework as to how social and cultural aspects of scientific creativity actually underlie creative cognition. As a result, teaching practices are not culturally adapted to foster creative cognition. The challenge is therefore to optimally integrate arts in STEAM education, to reach educational goals.

One field of particular interest to explore STEAM education is the domain of space. The space industry evolved through international collaboration, interdisciplinarity, and innovative thinking. Many recognize the attraction that space has on learners. According to motivation theory, students are most creative when they are intrinsically motivated through interest, enjoyment, satisfaction, and challenge of the work itself (Amabile, 1996; Amabile and Fisher, 2000; Hennessey et al., 2015). Intrinsic motivation is related to deep learning as well (Vansteenkiste et al., 2006). Thus, the interest inducing, and imaginative domain of space represents an appropriate context to foster the creative aspect of STEAM education (see Annex 1).

Since 1999, the World Space Week (United Nations for Outer Space Affairs) encourages STEAM education. Other examples are “The Space for STEAM” working group of the International Astronautical Association (IAA). A Team Project at the International Space University, 2012; Boy, 2013) mentioned that space-related content is excellent for STEAM education because it (1) inspires and motivates creativity, (2) is interdisciplinary, (3) appeals to both genders and promotes equality, (4) promotes international and cross-cultural cooperation, and (5) strives for a common and thriving future.

In the following paragraphs I address creative scientific cognition within the STEAM framework. Then, I elaborate on social and cultural aspects of scientific creativity and propose future research directions to inform teaching of STEAM. Throughout the paper I underscore the unique role of Space to foster STEAM education.

## Creativity and Steam: Creative Scientific Cognition

There are many domains of creativity, and maybe most relevant in the arts and sciences (Kaufman and Baer, 2006). Science is a creative field of work, including when students find and solve scientific problems (Sternberg et al., 2020). Scientific creativity can be defined as any thought or behavior in science that is both novel and useful (e.g., Feist, 2011; Cropley, 2015; De Vries, 2018).

Within the multivariate approach to creativity, it is thought that different factors are involved in creative performance, such as knowledge, cognitive style, motivation, emotions, personality, and environment (e.g., culture and context) (Sternberg and Lubart, 1995). There are multiple approaches to investigate creativity, such as with neuroscience to discover mechanisms underlying cognition (e.g., Benedek and Fink, 2019; Khalil et al., 2019), psychological research (e.g., De Vries, 2018), and also qualitative approaches (e.g., Moran et al., 2003).

Kaufman and Beghetto (2009) discern different levels of creative expression: eminent creativity (C- creativity), professional-level expertise (Pro-c), everyday creativity (little-c) and personal creative expression as inherent in the learning process (mini-c). Within the educational field, and STEAM framework, the focus is on mini-c and how the cognitive process of students is related to the contextual factor of the educational environment.

Since Guilford's renowned presentation at APA on creativity 1950, specifying the divergent and convergent process of creativity, many more processes of creativity have been analyzed (e.g., Sawyer, 2011). Interestingly, for scientific creativity, the two-step process is often maintained as in the divergent exploratory and convergent integrative “Dual Search Model” model of Klahr and Dunbar (1988). However, all activities of science from hypothesis formation, testing, evaluating results, to writing results, are related to creativity. Today there are only few tests to assess creative scientific thinking of younger students, such as the Scientific Ability Test (C-SAT) (Ayas and Sak, 2014), and the Evaluation of Potential Creativity (EPoC) battery (Lubart et al., 2013).

There are cognitive skills which are particularly relevant for scientific creativity. Examples are the use of metaphors and analogies, which serve homospatial thinking, and janusian thinking, which represents more spatial and simultaneous cognition of two opposite thoughts. Other examples of cognitions are linear and non-linear thinking, sepconic articulation processes, associations, dialectical synthesis, and synthesis of ideas and bi-sociation (e.g., Koestler, 1964; Tweney, 1996; Groves et al., 2008; Feist, 2011; Rothenberg, 2011).

Thus, we might ask how does the integration of art in STEAM relate to underlying cognitive processes? Authors such as Kim et al. (2012), and Miller and Knezek (2013) argue that even today there is a lack of conceptualization of STEAM, in that it consists of simply “adding the arts.” From a pedagogical perspective, art in STEAM relates to different concepts, such as plural “arts” to mean the liberal arts, whereas the singular “art” refers to visual, musical, and performance art, and mathematics. Delaney (2014) specifies that the ultimate goal of this model is to explore and articulate criteria of STEAM-based practices, such as problem-based delivery, discipline integration, problem-solving skills, instructional approaches, assessment practices, and equitable participation. A second question could therefore be, do teacher practices sufficiently aim at fostering creative cognitive processes?

Yakman (2008) defines the arts as going beyond aesthetics and includes the liberal arts relating the subjects through interdisciplinary approaches. Her well-known “STEAM Framework for Education Across the Disciplines,” implies higher-level synthesis producing holistic, integrative knowledge, and includes “key elements” of arts pertaining to the different STEM disciplines. The STEAM framework is not clear on how ‘key elements” of arts relate to higher level synthesis in scientific thinking across disciplines. As a consequence, the framework does not address creative, social, or cultural aspects involved in higher synthesis. I propose that research on scientific creativity can fill this gap and foster STEAM education.

A more granular facet of cognition involved in scientific creativity, is the analysis of conceptual combinations (Ward et al., 2002). This is related to the research field of conceptual change (Carey, 2009; Vosniadou, 2009). Knowledge acquisition in science is also related to this domain. It is thought that science learning involves either a gradual addition, elimination, and organization of concepts, or a revolutionary process, where one theory of conceptual understanding is replaced with another.

Creative scientific cognition represents at the core where the STEAM framework fosters creative thinking in STEM through the arts. The interdisciplinary teaching moreover enhances scientific creative thinking, as it for example promotes the synthesis and integration of previously unconnected concepts.

Scientific creativity is also fostered by broadening boundaries of scientific concepts. Mentions that there is a social aspect involved in the breaking down, and creation and reformulation of boundaries. Consider a remark of Russian cosmonaut Sergey Ryazanskiy (2020): “*Before my flight I realized there were many borders and boundaries we created in ourselves and in our lives…After working from our planet of above you understand that there are no visible borders, all these borders and boundaries we create ourselves in our mind. If we understand this, we will be able to do much more than we ever can imagine.”* This example of the experience of space indicates that social and cultural aspects of the space endeavor are related to broadening of concepts, and therefore creative cognition.

In summary, the “key elements” of the integration of arts in the STEAM framework pertain to creative cognition and its social and cultural aspects. I now turn to these social and cultural factors to elaborate on how they relate to creative scientific cognition.

## Scientific Creativity: Social and Cultural Factors

The domain of space is an intercultural and international endeavor that concerns all sciences and represents therefore a suitable domain, to explore cultural factors related to STEAM and scientific creative cognition.

The research domain of cultural differences in creativity is growing (Lubart et al., 2019). Most research on cultural differences of creativity compare levels of creativity for adult populations, such as for divergence and convergence (Cheung et al., 2016), or the influence of multicultural experience. Kharkhurin (2012) for example found that multiculturalism and multilingualism were related to enhanced creative potential. He theorized that the encounter with other cultures enhances flexible thought. Leung et al. (2008) showed that for adults, multi-cultural experience relates to cognitive processes supporting creativity through the use of unconventional knowledge and ideas of unfamiliar. Other explanations are that encountering others culture causes the expansion of ideas, such that retrieving concepts of two or more cultures and integrating them causes new insights (Wan and Chiu, 2002). Simonton (2000) related bi- or multiculturalism to cognitive processes of “novel conceptual combinations,” resulting in creative conceptual expansion.

However, other findings indicate that the relation between culture and creativity is more complex. Empirical research on first year college students (de Vries et al., 2015) found that multicultural experience could also impede creativity for students with specific cultural backgrounds, contrary to the general findings. Other studies (de Vries and Lubart, 2017) with younger students, also found that students with immigrant cultural backgrounds had a reduced capacity of synthesizing and integration of concepts for scientific creativity, which impeded creativity. These findings underscore the importance of understanding cultural factors of STEAM education.

The “Cultural Actuation Model” (De Vries, 2018), is based on a study with young students (ages 9 and 10) from India, Russia, and Europe. Different cultural environments are more or less conducive to kinds of creativity. In this model, the attitude of “Tolerance of Ambiguity and Uncertainty”(TA) (Frenkel-Brunswik, 1949) and the cultural value of ‘Power distance”(PD) (Hofstede, 2011), were related to students producing ideas based on observable, “surface” features, “process” oriented features, or ideas based on abstract, or “core” features. Ideas based on observable features, mostly related to low-TA and high-PD environments, were found to be less creative than ideas containing “process” and “core” features.

The particularity of this research model is that it focussed on cultural differences of features or patterns, instead of levels of creativity. It is also in line with a “warming trend” of conceptual change research, away from “cold conceptual change.” This means that there is a growing focus on social, motivational, contextual, affective factors, and background knowledge of learners (Vosniadou, 2009).

Overall, more research is needed to understand and confirm the role of social and cultural factors on scientific creativity. Future studies could also focus on different stages during development in relation with the impact of social and cultural factors. Cross-cultural research could focussing on culturally varying patterns of creative productions and scientific creative cognition. It is possible that adding different cultures will reveal unknown aspects.

Teaching practices are also related to social and cultural factors. This raises the question what practices foster or maybe impede scientific creative cognition. This is discussed in the following paragraph.

## Teaching for Steam: Cultural Practices and Creative Scientific Cognition

Despite interest from governments and the educational environment for STEAM education (Henriksen, 2014), less is known about cultural differences in teacher practices, or how STEAM is implemented in different cultures. Teachers practices, while using the same educational tool, can differ, and this could be critical. Studies exploring how cultural differences influence teaching for STEAM is an emerging field (Yakman and Lee, 2012). Effects of teacher's roles and practices in general on learning outcomes, however, are not well known.

In contrast to this gap in research, teacher's intercultural competence is becoming more important because of today's increasingly multicultural classrooms. Culturally sensitive teaching mostly focusses on topics such as language choice, religion equality, or culture courses for students (Rengi and Polat, 2019). There is a focus on intercultural sensitivity as an orientation which can for example be ethnocentric, transitional, or ethno-relative (Kuusisto et al., 2015). Others again address the gap in relationships between teachers and culturally diverse students and as a lack of care (Thapa, 2020).

The question can be asked if certain teacher practices are better suited to foster creative cognition. In their annual report, “The World Economic Forum” found that a “copy and paste” method of implementing best teaching practices across cultures was not possible. This was measured according to a ranking of learned cognitive skills of different countries [(Learning Curve Data Bank (LCDB), 0000)]. Wursten and Jacobs (2013) suggest that the problem is the unknown link of what happens between measurable “inputs” (funds, years of schooling, teacher-student ratio's, etc.) and “outputs” as learned outcomes, and therefore could be compared to a “black box.” The authors propose that the “input” of cultural context needs to be analyzed and that teacher's practices should be adapted to cultural contexts. Cultural values are deeply embedded and entangled with social and educational policies. For example “right” behavior of a student in one culture, could be “wrong” behavior in another. They summarize examples of implications of attitudes according to cultural values of teachers, and students.

To gain insight into the “black box,” future research could therefore focus on culturally different teacher practices, and student and teacher attitudes, related to value dimensions. The next step would then be to measure learning outcomes of specific aspects of scientific creative cognition. The relation of the teacher practices and learning outcomes will reveal how to foster best scientific creative processes.

To demonstrate the creativity challenge of this paper, Annex 2 shows an illustrative example (De Vries, 2018) which differentiates student and teacher attitudes and practices according to TA and the PD value dimension, and from culture and creativity literature (Hofstede, 2001; Sawyer, 2011). Possible related learning outcomes of creative cognition are mentioned, based on results of the previously mentioned study. In this way, research could relate teacher practices to learning outcomes, by integrating qualitative analysis of teacher and student attitudes and teacher practices, as well as quantitative measurement of creative scientific cognition.

In sum, we need to understand how cultural differences in teacher practices fosters different aspects of creative cognition. Future directions of research could (1) investigate further social and cultural effects of creative cognition, (2) analyze cultural differences of classroom organizations, to understand how constellations of cultural values “work out” in classroom practices, and (3) assess how practices are related to differences in learned outcomes of scientific creative cognition.

Finally, other “layers” of culture such as gender, and socio-economic backgrounds should also be addressed. Space fits this new direction of research because of the international collaboration in this domain, which offers possibilities for international collaboration on cross-cultural STEAM education, as well as opportunities to exchange teacher practices. The ultimate challenge is that all students, regardless of their cultural backgrounds, can fully develop their scientific creative cognition.

## Conclusion

It was argued that a new challenge in education of “Space for STEAM” is a greater understanding of how cultural differences of teaching practices impact learning outcomes of scientific creative cognition. This is closely related to gaining an in-depth knowledge on social and cultural factors of creative cognition itself.

By broadening the STEAM education framework by integrating the empirical domain of scientific creativity, the Arts component is no longer “simply added,” but forms an essential part to increase scientific creative cognition and innovative thinking.

Practical implications are for example that teacher's STEAM education could target specific scientific creative cognitive processes. Another example is that results can inform STEAM teacher curricula, and training, to enhance intercultural teaching competence of teachers, specifically for cultural differences in practices as related to creative cognitive learning outcomes. This could result in fostering higher levels of scientific creative cognition of students of all cultures. It could be that certain teacher practices foster certain aspects of creative cognition more than others. If these are known, intercultural exchange can improve teaching. If these practices remain undiscovered however, a “harmonizing” of teacher practices for example by “copy and pasting” them, could risk reducing, instead of enhancing, learning outcomes.

Although there is emerging knowledge on cultural differences in teaching practices of STEAM, as well as on cultural factors of scientific creative cognition, more research is needed to predict further implications for optimal STEAM education.

The need for innovative scientific thinking makes it inevitable to take up this challenge in the foreseeable future. The naturally innovative, intercultural collaborative, and interdisciplinary aspects of the space domain, are as crucial for problem solution finding and sustainability in outer space as on planet earth.

## Author Contributions

The author confirms being the sole contributor of this work and has approved it for publication.

## Conflict of Interest

The author declares that the research was conducted in the absence of any commercial or financial relationships that could be construed as a potential conflict of interest.

## References

[B1] Amabile T. M. (1996). Creativity in Context. Boulder, CO: Westview Press.

[B2] Amabile T. M. Fisher C. M. (2000). Stimulate creativity by fueling passion, in Handbook of Principles of Organizational Behavior, ed Locke E. (West Sussex: John Wiley & Sons), 481–497.

[B3] Ayas M. B. Sak U. (2014). Objective measure of scientific creativity: psychometric validity of the Creative Scientific Ability Test. Think. Skills Creat. 13, 195–205. 10.1016/j.tsc.2014.06.001

[B4] Bae J. H. So K. H. Yun B. H. Kim J. S. Han G. I. Kim S. G.. (2014). The effects of science lesson applying STEAM education on creative thought activities and emotional intelligence of elementary school students. J. Korean Elementary Sci. Educ. 33, 762–772. 10.15267/keses.2014.33.4.762

[B5] Barlex D. Pitt J. (2000). Interaction: The Relationship Between Science and Design and Technology in the Secondary School Curriculum. Engineering Council.

[B6] Benedek M. Fink A. (2019). Toward a neurocognitive framework of creative cognition: the role of memory, attention, and cognitive control. Curr. Opin. Behav. Sci. 27, 116–122. 10.1016/j.cobeha.2018.11.002

[B7] Boy G. A. (2013). From STEM to STEAM: toward a human-centred education, creativity and learning thinking, in Proceedings of the 31st European Conference on Cognitive Ergonomics (New York, NY), 7. 10.1145/2501907.2501934

[B8] Carey S. (2009). The origin of concepts. New York, NY: Oxford University Press.

[B9] Cheung P. C. Lau S. Lubart T. Chu D. H. Storme M. (2016). Creative potential of Chinese children in Hong Kong and French children in Paris: a cross-cultural comparison of divergent and convergent-integrative thinking. Think. Skills Creat. 22, 201–211. 10.1016/j.tsc.2016.09.005

[B10] Cropley D. H. (2015). Promoting creativity and innovation in engineering education. Psychol. Aesthet. Creat. Arts 9:161. 10.1037/aca0000008

[B11] De Boer E. G. (1991). A History of Ideas in Science Education. New York, NY: Teachers College Press.

[B12] De Vries H. (2018). Cultural Differences of Scientific Creativity: a Relation with Tolerance of Ambiguity/Uncertainty: an Empirical Study With Children in Luxembourg, France, Thailand, India, and Russia (Ph.D. dissertation). Sorbonne Paris Cité. Paris, France. Available online at: http://www.theses.fr/2018USPCB052 (accessed October 1, 2020).

[B13] de Vries H. Kirsch C. J. Furnham A. (2015). Cultural differences in creativity: the role of immigration. Int. J. Talent Dev. Creat. 2, 41–51.

[B14] de Vries H. B. Lubart T. I. (2017). Scientific creativity: divergent and convergent thinking and the impact of culture. J. Creat. Behav. 53, 145–155. 10.1002/jocb.184

[B15] Delaney M. (2014). Schools Shift From STEM to STEAM. EdTech, 1–4. Available online at http://www.edtechmagazine.com/k12/article/2014/04/schools-shift-stem-steam

[B16] Feist G. (2011). Creativity in science, in Encyclopedia of Creativity, Vol. 2, eds Runco M. A. Pritzker S. R. (London: Elsevier), 296–302. 10.1016/B978-0-12-375038-9.00192-8 (accessed October 1, 2020).

[B17] Frenkel-Brunswik E. (1949). Intolerance of ambiguity as an emotional and perceptual personality variable. J. Personal. 18, 108–143. 10.1111/j.1467-6494.1949.tb01236.x 4833114

[B18] Groves K. Vance C. Paik Y. (2008). Linking linear/nonlinear thinking style balance and managerial ethical decision-making. J. Business Ethics 80, 305–325. 10.1007/s10551-007-9422-4

[B19] Guilford J. P. (1950). Creativity. Am. Psychol. 5, 444–454. 10.1037/h0063487 14771441

[B20] Harris A. de Bruin L. (2017). STEAM Education: fostering creativity in and beyond secondary schools. Aust. Art Educ. 38:54.

[B21] Hennessey B. Moran S. Altringer B. Amabile T. M. (2015). Extrinsic And Intrinsic Motivation. inWiley Encyclopedia Management, ed Cooper C. L. (Hoboken, NJ: John Wiley. & Sons), 1–2. 10.1002/9781118785317.weom110098

[B22] Henriksen D. (2014). Full STEAM ahead: creativity in excellent STEM teaching practices. STEAM J. 1:15. 10.5642/steam.20140102.15

[B23] Hofstede G. (2001). Culture's Consequences: Comparing Values, Behaviors, Institutions and Organizations Across Nations. Berkeley, CA: Sage Publications.

[B24] Hofstede G. (2011). Dimensionalizing cultures: the Hofstede model in context. Online Read. Psychol. Cult. 2, 2307–0919. 10.9707/2307-0919.1014

[B25] International Space University (2012). Team Project Final Report: Space one Giant Leap for Education. Retrieved from https://isulibrary.isunet.edu/doc_num.php?explnum_id=413

[B26] Kaufman J. C. Baer J. (2006). Creativity Research in English-Speaking Countries. Cambridge: Cambridge University Press.

[B27] Kaufman J. C. Beghetto R. A. (2009). Beyond big and little: the four c model of creativity. Rev. Gen. Psychol. 13, 1–12. 10.1037/a001368833391115

[B28] Keys C. W. Bryan L. A. (2001). Co-constructing inquiry-based science with teachers: essential research for lasting reform. J. Res. Sci. Teach. 38, 631–645. 10.1002/tea.1023

[B29] Khalil R. Godde B. Karim A. A. (2019). The link between creativity, cognition, and creative drives and underlying neural mechanisms. Front. Neural Circuit 13:18. 10.3389/fncir.2019.00018 30967763 PMC6440443

[B30] Kharkhurin A. V. (2012). Multilingualism and Creativity. Bristol: Multilingual Matters. 10.21832/9781847697967

[B31] Kim S. W. Chung Y. L. Woo A. J. Lee H. J. (2012). Development of a theoretical model for STEAM education. J. Korean Assoc. Sci. Educ. 32, 388–401. 10.14697/jkase.2012.32.2.388

[B32] Klahr D. Dunbar K. (1988). Dual space search during scientific reasoning. Cogn. Sci. 12, 1–48. 10.1207/s15516709cog1201_1

[B33] Koestler A. (1964). The Act of Creation. New York, NY: Penguin Books.

[B34] Kuusisto E. Kuusisto A. Rissanen I. Holm K. Tirri K. (2015). Finnish teachers' and students' intercultural sensitivity. J. Relig. Educ. 63, 65–77. 10.1007/s40839-016-0018-0

[B35] Learning Curve Data Bank (LCDB) Economist Intelligence Unit. Retrieved from https://eiuperspectives.economist.com/talent-education/learning-curve-2014.

[B36] Leung A. K. Y. Maddux W. W. Galinsky A. D. Chiu C. Y. (2008). Multicultural experience enhances creativity: the when and how. Am. Psychol. 63:169. 10.1037/0003-066X.63.3.169 18377107

[B37] Lubart T. Glaveanu V. de Vries H. Camargo A. Storme M. (2019). Cultural perspectives on creativity, in The Cambridge Handbook of Creativity, 2nd Edn, eds Kaufman J. C. Sternberg R. J. (New York, NY: Cambridge University Press), 421–447. 10.1017/9781316979839.022

[B38] Lubart T. I. Besançon M. Barbot B. (2013). Evaluation du potentiel créatif (EPoC). Paris: Editions Hogrefe France.

[B39] Lunar Games (NASA HQL-433 7-96) (2004). Available online at: https://er.jsc.nasa.gov/seh/moongame.html

[B40] Miller J. Knezek G. (2013). STEAM for student engagement, in Society for Information Technology and Teacher Education International Conference (Waynesville, NC), 3288–3298. (accessed October 1, 2020).

[B41] Moran S. John-Steiner V. Sawyer R. (2003). Creativity in the making. Creat. Dev, 61–90. 10.1093/acprof:oso/9780195149005.003.0003

[B42] OECD Education 2030; UN 2030 Global Goals for Sustainable Development (SDGs). (2018). The Future of Education and Skills Education 2030. Retrieved from: http://www.oecd.org/education/2030-project/contact/E2030_Position_Paper_(05.04.2018).pdf

[B43] Rengi Ö. Polat S. (2019). Practices related to cultural diversity in schools and students' views about these practices: Stuttgart, Baden-Württemberg, Germany. Egitim ve Bilim 44:197. 10.15390/EB.2019.7613

[B44] Rothenberg A. (2011). Janusian, homospatial and sepconic articulation processes, Encyclopedia of Creativity, eds Pritzker S. R. Runco M. A. (New York, NY: Academic Press), 1–9. 10.1016/B978-0-12-375038-9.00128-X

[B45] Sanders M. Kwon H. Park K. Lee H. (2011). Integrative STEM (Science, Technology, Engineering, and Mathematics) education: contemporary trends and issues. Secondary Educ. Res. 59, 729–762. 10.25152/ser.2011.59.3.729

[B46] Saptono S. Hidayah I. (2020). Scientific creativity: a literature review. J. Phys Conf. Ser. 1567:022044. 10.1088/1742-6596/1567/2/022044

[B47] Sawyer R. K. (2011). Explaining Creativity: The Science of Human Innovation. New York, NY: Oxford University Press.

[B48] Sergey Ryazanskiy (2020). Interview. Retrieved from https://www.youtube.com/watch?v=Si75wY9QC-g

[B49] Simonton D. K. (2000). Creativity: cognitive, personal, developmental, and social aspects. Am. Psychol. 55, 151–158. 10.1037/0003-066X.55.1.151 11392859

[B50] Sternberg R. J. Lubart T. I. (1995). Defying the Crowd: Cultivating Creativity in a Culture of Conformity. New York, NY: Free Press.

[B51] Sternberg R. J. Todhunter R. J. Litvak A. Sternberg K. (2020). The relation of scientific creativity and evaluation of scientific impact to scientific reasoning and general intelligence. J. Intell. 8:17. 10.3390/jintelligence8020017 32326475 PMC7713009

[B52] Thapa S. (2020). Assessing intercultural competence in teacher education: a missing link, in Visions for Intercultural Music Teacher Education (Cham: Springer), 163–176. 10.1007/978-3-030-21029-8_11

[B53] Tweney R. D. (1996). Presymbolic processes in scientific creativity. Creativity Res. J. 9, 163–172. 10.1207/s15326934crj0902

[B54] Vansteenkiste M. Lens W. Deci E. L. (2006). Intrinsic versus extrinsic goal contents in self-determination theory: another look at the quality of academic motivation. Educ. Psychol. 41, 19–31. 10.1207/s15326985ep4101_4

[B55] Vosniadou S. (Ed.). (2009). International Handbook of Research on Conceptual Change. New York, NY: Routledge. 10.4324/9780203874813

[B56] Wan W. W. Chiu C. (2002). Effects of novel conceptual combination on creativity. J. Creat. Behav. 36, 227–240. 10.1002/j.2162-6057.2002.tb01066.x

[B57] Ward T. B. Patterson M. J. Sifonis C. M. Dodds R. A. Saunders K. N. (2002). The role of graded category structure in imaginative thought. Memory Cogn. 30, 199–216. 10.3758/bf03195281 12035882

[B58] Wursten H. Jacobs C. (2013). The impact of culture on education. Can we introduce best practices in education across countries. ITIM Int. 1, 1–28.

[B59] Yakman G. (2008). STEAM education: an overview of creating a model of integrative education, in Pupils' Attitudes Towards Technology (PATT-19) Conference: Research on Technology, Innovation, Design and Engineering Teaching (Salt Lake City, UT).

[B60] Yakman G. Lee H. (2012). Exploring the exemplary STEAM education in the US as a practical educational framework for Korea. J. Korean Assoc. Sci. Educ. 32, 1072–1086. 10.14697/jkase.2012.32.6.1072

