# Supplementary material for: Space for STEAM: New Creativity Challenge in Education
Source: Front Psychol. 2021 Mar 23;12:586318. doi: 10.3389/fpsyg.2021.586318 (PMC8025669; doi:10.3389/fpsyg.2021.586318)
Supplement: Supplementary file 1 [file Data_Sheet_1.docx]

**Annex 1**

**Space for STEAM Educational Tool: ‘Lunar Games’**


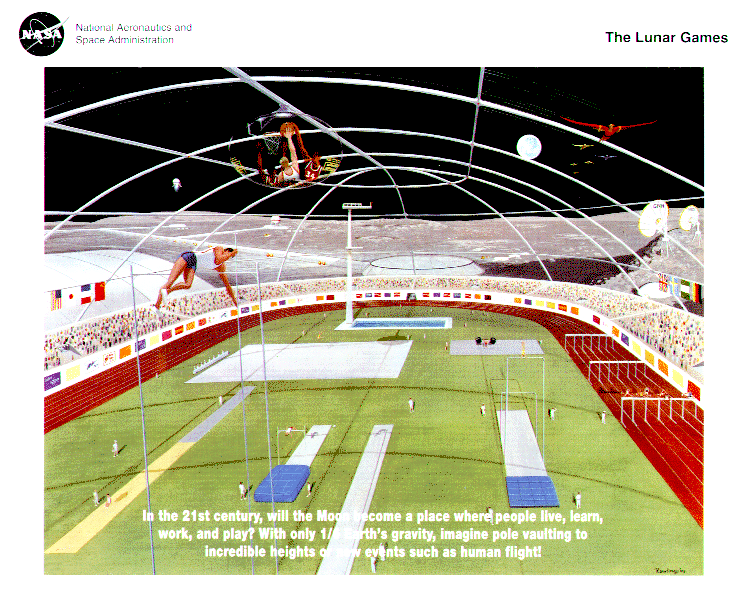


Fig: ‘The Lunar Games’ (Patrick Rawling, 1996)

An example of an educational tool to promote Space for STEAM, is invented through a collaboration of S. Brodry and painter Patrick Rawlings. The athletic event of 'Olympic proportions' portrays a vision with the explicit intent to stretch the imagination for humans in space well beyond the ‘normal’ of government-funded astronauts exploring another world. It is based on an original work of art, originally called by the artist “A Leap of Faith”, and was subsequently named “The Lunar Games”. A two-sided lithograph of it was mass-produced and used throughout the United States for education and public outreach purposes. Many were also disseminated to those visiting Atlanta (USA) during the 1996 Olympics there, when NASA hosted a Space Station mock-up exhibit at a nearby public venue. (NASA HQL-433, 7-96; <https://er.jsc.nasa.gov/seh/moongame.html>

“The Lunar Games” is widely used in lectures and outreach to school classrooms at the primary, secondary, and university levels. <https://er.jsc.nasa.gov/seh/moongame.html>)

The topic motivates students interested in sports at any level, and it inspires creative thought about what the future may hold for humanity and for each individual. Whereas the main objective is to enhance creative thinking in STEM subjects, many students also develop an interest in creative arts themselves (e.g., painting, literature, theatre, and other modes of communication) melded with the space field and its rich imagery. Space for STEAM education thus fosters interest in opportunities of employment for those who do not pursue STEM academic backgrounds or careers directly in STEM subject areas.

Students are asked to find scientific solutions in the extreme environment of the moon’s atmosphere.

Not only are some of the competitions depicted as appropriately ‘super-sized’ -- such as pole vaulting to fantastic heights, given that there is only 1/6 Earth’s gravity on the Moon -- the artist also portrays human-powered flight, an athletic event conceivably made possible in the ‘extreme environment’ of our celestial companion.

The use of artistic talents to convey space-related images, themes, and stories of course is not new; science fiction in literature, film, and TV creations reflect the long-standing bond between space and the arts. But students who see these presentations witness their power to instill what some have called a transcendent or ‘spiritual moment’, sometimes also a coming-together across disciplines, philosophies, and cultural backgrounds. Such ‘encounters’, outside one’s normal experiences, can provide fertile ground for transformative and creative thought, action, and performance, as well as for intercultural learning and dialogue. For all students, the wonderment of space and realization of human connection to “the heavens above” ‘Space for STEAM’ represents an effective educational tool.
