# Supplementary material for: Space for STEAM: New Creativity Challenge in Education
Source: Front Psychol. 2021 Mar 23;12:586318. doi: 10.3389/fpsyg.2021.586318 (PMC8025669; doi:10.3389/fpsyg.2021.586318)
Supplement: Supplementary file 2 [file Data_Sheet_2.docx]

**Annex 2**

**Cultural Implications for Teaching and Learning (Illustrative Example in de Vries, H. (2018))**

| **Classroom Culture** | **Student’s Perspective** | **Teacher’s Perspective** | **Practices** | **Learned Outcome** |
| --- | --- | --- | --- | --- |
| **I**  Low level Tolerance for Ambiguity/  Uncertainty  High level of Power Distance | ‘In this class I need to pay attention to obeying the teacher, then the teacher is very happy with me and I will get good grades. I don’t need to think, but I cannot dream too much either, then the teacher will notice it’ | ‘A child needs foremost, to feel safe, to have structure.  ‘Discipline helps to learn well. The classroom environment also needs to be well organized and tidy.’ | - repetition  - exercises with emphasis is on copying | Low creative cognition:  Low fluency and Low originality  (surface ideas) |
| **II**  Low level Tolerance for Ambiguity/  Uncertainty  Medium level of Power Distance | ‘In this class, I need to obey and listen well to the teacher. I know if I actively participate in lessons, I get good grades. I know the teacher observes me how I carry out a task or when we do group work, but he/she does not understand or know what I am thinking’ | ‘I pay attention to the method of teaching, if children follow this, and carry out a task the right way. When children are disciplined and show effort, they will do well’ | - repetition  - surface exercises  - sometimes group work  - deductive reasoning | Medium creative cognition:  Low fluency and Medium originality  (surface and process ideas) |
| **III**  High level Tolerance for Ambiguity/ Uncertainty  Medium level of Power Distance | ‘In this class, things are more relaxed. I can move freely around, even sit on the floor (or maybe the teachers chair) if I feel like it. I know the teacher doesn’t judge me so much, but I do need to show I am also really working for myself and understand what the lesson is about and what we are learning’ | ‘I want children to feel good in their class, so that they can express their own way of thinking, their own ideas, this will help them learn.’  ‘It is normal that children don’t sit still and that the class is not orderly, I look at the result’ | - open ended questions  - minimal organization  - inductive reasoning | Medium creative cognition:  High fluency and  Medium originality  (process and core ideas) |
| **IV**  High level Tolerance for Ambiguity/  Uncertainty  Low level of Power Distance | ‘In this class I need to be fully active: the teacher expects me to participate and ask questions, follow the lesson (but somehow it is fun), listen to the rules (which we made together), and I know the teacher also wants me to really think for myself, children in this class come up with their own ideas, the teacher appreciates this’ | ‘I am focused on the child’s thinking, and when children feel that there are sensible rules which are not too strict, and the way I teach is engaging, they will learn to express their own way of thinking’ | - sometimes repetition  - engaging activities  - choice in exercises  - sometimes group work.  - individual thinking exercises | High creative cognition  High Fluency and  High Originality  (surface and process and core ideas) |

De Vries, H. (2018) Cultural differences of scientific creativity: a relation with tolerance of ambiguity and uncertainty: an empirical study with children in Luxembourg, France, Thailand, India, and Russia. Doctoral dissertation. Sorbonne Paris Cité. Paris, France. <http://www.theses.fr/2018USPCB052>
